# Different Prognostic Values of Dual-Time-Point FDG PET/CT Imaging Features According to Treatment Modality in Patients with Non-Small Cell Lung Cancer

**DOI:** 10.3390/tomography8020087

**Published:** 2022-04-08

**Authors:** Su Jin Jang, Jeong Won Lee, Ji-Hyun Lee, In Young Jo, Sang Mi Lee

**Affiliations:** 1 Department of Nuclear Medicine, CHA Bundang Medical Center, CHA University, 59 Yatap-ro, Bundang-gu, Seongnam 13496, Korea; jsjnm07@cha.ac.kr; 2 Department of Nuclear Medicine, International St. Mary’s Hospital, Catholic Kwandong University, Simgok-ro 100 gil 25, Seo-gu, Incheon 22711, Korea; jwlee223@ish.ac.kr; 3 Department of Pulmonology, Allergy and Critical Care Medicine, CHA Bundang Medical Center, CHA University, 59 Yatap-ro, Bundang-gu, Seongnam 13496, Korea; plmjhlee@cha.ac.kr; 4 Department of Radiation Oncology, Soonchunhyang University Cheonan Hospital, 31 Suncheonhyang 6 gil, Dongnam-gu, Cheonan 31151, Korea; inyoung.jo@schmc.ac.kr; 5 Department of Nuclear Medicine, Soonchunhyang University Cheonan Hospital, 31 Suncheonhyang 6 gil, Dongnam-gu, Cheonan 31151, Korea

**Keywords:** non-small cell lung cancer, F-18 fluorodeoxyglucose, positron emission tomography, prognosis

## Abstract

This study was aimed to investigate whether dual-time-point F-18 fluorodeoxyglucose (FDG) positron emission tomography (PET)/computed tomography (CT) imaging features had different prognostic values according to the treatment modality in patients with non-small cell lung cancer (NSCLC). We retrospectively reviewed 121 NSCLC patients with surgical resection (surgery group) and 69 NSCLC patients with chemotherapy and/or radiotherapy (CRT group), who underwent pretreatment dual-time-point FDG PET/CT. The maximum standardized uptake value (SUV), metabolic tumor volume (MTV), total lesion glycolysis (TLG), SUV histogram entropy of primary cancer, and the percent changes in these parameters (Δparameters) were measured. In multivariate analysis, MTV, TLG, and entropy on both early and delayed PET/CT scans were significantly associated with progression-free survival (PFS) in the surgery group, but all Δparameters failed to show a significant association. In the CRT group, TLG on the early PET, maximum SUV on the delayed PET, ΔMTV, and ΔTLG were significant independent predictors for PFS. In the surgery group, patients with high values of MTV, TLG, and entropy had worse survival, whereas, in the CRT group, patients with high values of ΔMTV and ΔTLG had better survival. Dual-time-point FDG PET/CT parameters showed different prognostic values between the surgery and CRT groups of NSCLC patients.

## 1. Introduction

Non-small cell lung cancer (NSCLC) comprises over 80% of all lung cancers [1]. For NSCLC patients without distant metastasis, surgical resection is considered a potentially curative treatment and, in inoperable cases among them, radiotherapy with or without chemotherapy is recommended as the standard of care [2]. Despite the progress in management strategies in recent decades, NSCLC is still notable for its poor prognosis. Over 30% of patients with stage I–III disease who had received surgical resection experienced cancer recurrence and, in patients with unresectable stage III, the median overall survival was only 12.9 months [3,4]. Therefore, several studies have assessed various clinical factors as prognostic factors that could predict clinical outcomes and aid in selecting the appropriate treatments [5,6]. In addition to the well-established prognostic factors of NSCLC including the TNM stage, recent studies found several prognostic factors related to the biological characteristics of the tumors such as genetic mutations in cancer cells and intratumoral heterogeneity [6,7]. Furthermore, for NSCLC patients treated with radiotherapy and chemotherapy, several studies have focused on identifying biomarkers that reflect biological activity and the proliferation of cancer cells, which are known to be significantly related to the treatment response [8,9].

F-18 fluorodeoxyglucose (FDG) positron emission tomography (PET)/computed tomography (CT) is one of the diagnostic examinations most commonly recommended in guidelines for NSCLC [2]. FDG PET/CT has shown substantial clinical benefits in detecting lung cancer lesions as well as staging and predicting the prognosis of NSCLC patients [10,11]. FDG PET/CT constantly demonstrates high sensitivity for detecting malignant pulmonary lesions, but because FDG also accumulates in inflammatory pulmonary lesions, it has a limited specificity in differentiating lung cancer lesions from benign pulmonary lesions [10,12]. To overcome this limitation, the dual-time-point FDG PET/CT scanning method was introduced, which is comprised of early conventional imaging one hour after FDG injection and delayed imaging 2–3 h after the injection [10,12,13]. The concept of dual-time-point FDG PET/CT is based on the finding that the FDG uptake of malignant lesions reaches a peak approximately five hours after the injection, whereas the FDG uptake of benign lesions is known to decrease or not change upon delayed imaging [12,13]. This leads to an increase in the difference in FDG uptake between malignant and benign lesions on delayed scan images; thus, the diagnostic accuracy of FDG PET/CT for lung cancer could be enhanced by using the imaging parameters on delayed PET/CT and changes in the imaging parameters between early and delayed PET/CT scans [12,14,15]. Furthermore, because the increment of FDG uptake on delayed PET/CT is considered to be related to the biological activity of tumors, several studies have attempted to investigate the prognostic significance of dual-time-point PET/CT parameters for predicting the clinical outcomes of patients with NSCLC; however, inconsistent results have been reported between previous studies [16,17,18,19]. Taking into account the effect of the biological activity of NSCLC on the response to radiotherapy and chemotherapy, dual-time-point PET/CT parameters might have different prognostic values according to the treatment modality of patients with NSCLC, but this has yet to be demonstrated.

In the present study, we classified patients with NSCLC into two groups (those treated with surgery and those treated with radiotherapy and/or chemotherapy) and evaluated whether dual-time-point PET/CT parameters had different prognostic significance for predicting the disease progression of NSCLC in those two groups.

## 2. Materials and Methods

### 2.1. Study Population

We retrospectively reviewed the medical records of 470 patients who underwent dual-time-point FDG PET/CT for the diagnostic work-up of a pulmonary nodule and/or mass lesions between March 2014 and May 2020 at CHA Bundang medical center. Among them, a total of 190 patients were finally enrolled in the present study according to the following criteria: patients (1) who were histopathologically diagnosed with NSCLC, (2) who showed no distant metastasis on pretreatment imaging examinations (M0 stage), and (3) who received surgical resection, concurrent chemoradiotherapy, chemotherapy alone, or radiotherapy alone for the treatment. We excluded the patients (1) who received only supportive care or palliative treatment other than surgery, chemotherapy, and radiotherapy, (2) who had a previous history of malignant disease, (3) who were lost to follow-up within 12 months after the initial treatment without an event, or (4) who had NSCLC tumors with low FDG uptake inadequate for tumor delineation using Nestle’s adaptive thresholding method. All enrolled patients underwent pretreatment diagnostic work-up including physical examination, blood tests, contrast-enhanced chest CT, brain magnetic resonance imaging (MRI), and FDG PET/CT. Based on the results of the diagnostic examinations and clinical condition of the patient, surgical resection, concurrent chemoradiotherapy, chemotherapy, or radiotherapy was performed. After the initial treatment, clinical follow-up was conducted at regular intervals of every 3–6 months with contrast-enhanced chest CT. In patients who showed abnormal findings on the follow-up examinations, further imaging studies and/or histopathological assessments were performed to confirm disease progression. Based on the initial treatment modalities, all patients were categorized into two groups; patients who underwent surgical resection (surgery group) and those who underwent chemotherapy and/or radiotherapy (CRT group).

### 2.2. Dual-Time-Point FDG PET/CT

Dual-time-point FDG PET/CT was performed with a dedicated PET/CT scanner (Biograph mCT, Siemens Healthineers, Erlangen, Germany). FDG was supplied by the commercial supplier (DuchemBio Co., Ltd., Seoul, Korea). All patients were instructed to fast for at least six hours before the PET/CT scan and after the confirmation of a blood glucose level of <200 mg/dL, a dose of 5.18 MBq/kg of FDG was intravenously injected. The early PET/CT was performed 60 min after FDG injection from the skull base to the mid-thigh, and the delayed PET/CT of the chest region was performed 120 min after radiotracer injection. For both PET/CT scans, a non-contrast-enhanced CT was initially performed with 100 kVp and 40 mA, and subsequently, a PET scan was performed for 1.5 min in each bed position using the three-dimensional acquisition mode. The PET images were reconstructed on a 200 × 200 matrix using the iterative ordered subsets expectation-maximization algorithm with attenuation correction.

### 2.3. Imaging Analysis

**Figure 1:**
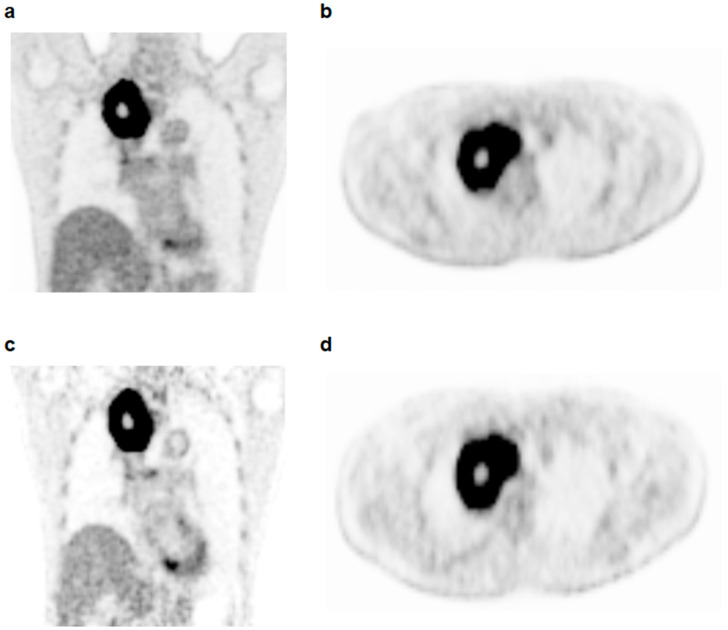
Coronal (**a**) and transaxial (**b**) images in early FDG PET/CT scans and coronal (**c**) and transaxial (**d**) images in the delayed FDG PET/CT scans of a 53-year-old man histopathologically diagnosed with adenocarcinoma. In both early and delayed PET/CT images, the primary lung cancer lesion was delineated using a threshold value of 7.06 for the early PET/CT images and 7.61 for the delayed PET/CT images determined by Nestle’s adaptive threshold method (**b**,**d**). The maximum SUV, MTV, and TLG were 21.9, 114.6 cm^3^, and 1218.2 g for early PET/CT and 26.9, 175.3 cm^3^, and 2236.8 g for delayed PET/CT. Therefore, the ΔMTV and ΔTLG were 53.0% and 83.6%, respectively. The patient was clinically diagnosed with T4N2M0 and received concurrent chemoradiotherapy. The patient had not experienced cancer progression during 32.7 months of follow-up.

The dual-time-point FDG PET/CT images were retrospectively assessed by two nuclear medicine physicians without knowing the clinical outcomes of the patients. The conventional parameters and first-order textural features of the primary lung cancer lesions were extracted from the PET images using LIFEx software version 7.0.0 (www.lifexsoft.org (accessed on 20 March 2021)) [20]. For each of the early and delayed PET images, a volume of interest (VOI) was manually drawn around the primary lung cancer lesion, and the delineation of primary lung cancer was performed using the tumor threshold calculated according to modified-Nestle’s adaptive thresholding method (Figure 1): (tumor threshold) = 0.3 × (tumor SUVmeanSUV70%) + (background SUVmean) [21,22,23]. The tumor SUVmeanSUV70% was calculated as the mean standardized uptake value (SUV) of all voxels surrounded by the isocontour set at 70% of the maximum SUV of the tumor within the VOI, and the background SUVmean was defined as the mean SUV of the background voxels [21]. The margins of the primary tumor lesions determined by the tumor threshold were manually inspected to avoid FDG uptake by the adjacent organs included within the primary tumor lesions. From the areas of the primary lung cancer lesions within the tumor threshold, four conventional PET parameters and four first-order PET textural features were extracted. The four conventional PET parameters were maximum SUV, mean SUV, metabolic tumor volume (MTV), and total lesion glycolysis (TLG), and the four first-order PET parameters were SUV histogram-based skewness, kurtosis, entropy, and energy (Table S1). Using the eight PET parameters measured from early and delayed PET images, the percent change in each PET parameter between the early and delayed PET images was calculated as follows: (ΔPET parameter) = [(parameter on delayed PET) − (parameter on early PET)]/(parameter on early PET) × 100. Therefore, there were a total of 24 dual-time-point PET parameters, comprised of eight parameters in early PET, eight parameters in delayed PET, and the percent change in eight parameters, for each patient.

### 2.4. Statistical Analysis

The baseline characteristics between the patient groups were compared using the Mann–Whitney test and the chi-squared test. The Wilcoxon signed-rank test was performed to evaluate the differences in the eight PET parameters between the PET scans. For survival analysis, the prognostic significance of the 24 dual-time-point PET parameters and the clinical factors in predicting progression-free survival (PFS) was assessed using univariate and multivariate Cox proportional hazards regression tests. PFS was defined as the time from the day of the initial treatment until the day of detection of disease progression or the day of the last follow-up visit. The dual-time-point PET parameters that showed statistical significance in univariate survival analysis were selected for multivariate analysis. In the multivariate survival analysis, the significance of the associations between the PET parameters and PFS was assessed by adding age, sex, and TNM stage as covariates for the analysis. Survival curves of the PET parameters were estimated using the Kaplan–Meier method to calculate cumulative PFS. For the Kaplan–Meier analysis, the specific cut-off values of the PET parameters were determined by receiver operating characteristic (ROC) curve analysis, and the patients were dichotomized according to the cut-off values. The statistical analyses were performed using MedCalc Statistical Software version 20.014 (MedCalc Software Ltd., Ostend, Belgium), and *p*-values of <0.05 were regarded as statistically significant.

## 3. Results

### 3.1. Patient Characteristics

Of the enrolled 190 patients, 121 patients received surgical resection (surgery group) and the remaining 69 patients were treated with chemotherapy and/or radiotherapy (CRT group). The baseline characteristics of the patients in both groups are shown in Table 1. The CRT group showed significantly higher proportions of patients with a smoking history and advanced tumor stage (*p* < 0.05). Among the patients in the surgery group, 63 patients (52.1%) received adjuvant treatment after surgery.

The duration of median follow-up for the enrolled patients was 28.3 months (range, 1.8–75.4 months). During follow-up, 95 patients (50.0%) were classified as having disease progression. There were 37 patients (30.6%) and 58 patients (84.1%) with disease progression in the surgery group and the CRT group, respectively. The patients in the CRT group revealed significantly worse survival (1-year PFS rate, 46.4% vs. 86.0%) than those in the surgery group (*p* < 0.001).

### 3.2. Comparisons of PET/CT Parameters

To assess the differences in the PET/CT imaging parameters between the early and delayed PET scans, the eight PET/CT parameters were compared pairwise (Table 2). All four conventional PET parameters on the delayed PET scan were significantly higher than those in the early PET scan (*p* < 0.001 for all), and for the maximum SUV, MTV, and TLG, over 90% of the patients revealed increased values on the delayed PET images. For the four first-order PET parameters, the delayed PET scans showed significantly increased skewness (*p* = 0.034) and entropy (*p* < 0.001) values compared to the early PET scans, whereas energy on the delayed PET scans was significantly lower than that on the early PET scans.

### 3.3. Survival Analysis

The prognostic significance of the 24 dual-time-point PET parameters for predicting PFS in univariate survival analysis is presented in Table 3, along with the clinical factors. In the surgery group, the maximum SUV, mean SUV, MTV, TLG, entropy, and energy in both the early and delayed PET scans were significantly associated with PFS (*p* < 0.05). However, none of the ΔPET parameters showed a significant association with PFS (*p* > 0.05). In the CRT group, the maximum SUV, mean SUV, MTV, and TLG in the early PET scans and the maximum SUV, mean SUV, and energy in the delayed PET scan were significant predictors of PFS (*p* < 0.05). In contrast to the results in the surgery group, ΔMTV, ΔTLG, and Δentropy were also significantly associated with PFS (*p* < 0.05). Among the clinical factors, T stage, N stage, and TNM stage showed significant associations with PFS in both the surgery and CRT groups (*p* < 0.05).

Among the 24 PET parameters, the parameters that showed a statistically significant association with PFS in the univariate analysis were included in the multivariate analysis with adjustment for age, sex, and TNM stage. The results of the multivariate analysis of the surgery group demonstrated that MTV, TLG, and entropy in both the early and delayed PET scans remained significant independent predictors of PFS (*p* < 0.05; Table 4). For all those six PET parameters, an increase in those PET parameter values was associated with an increased risk of disease progression.

In the CRT group, TLG on early PET, the maximum SUV on delayed PET, ΔMTV, and ΔTLG were significant independent predictors of PFS (*p* < 0.05; Table 5). For TLG on the early PET and maximum SUV on the delayed PET, an increase in values was associated with an increased risk of disease progression. Contrastingly, an increase in ΔMTV and ΔTLG was associated with a decreased risk of disease progression.

**Figure 2:**
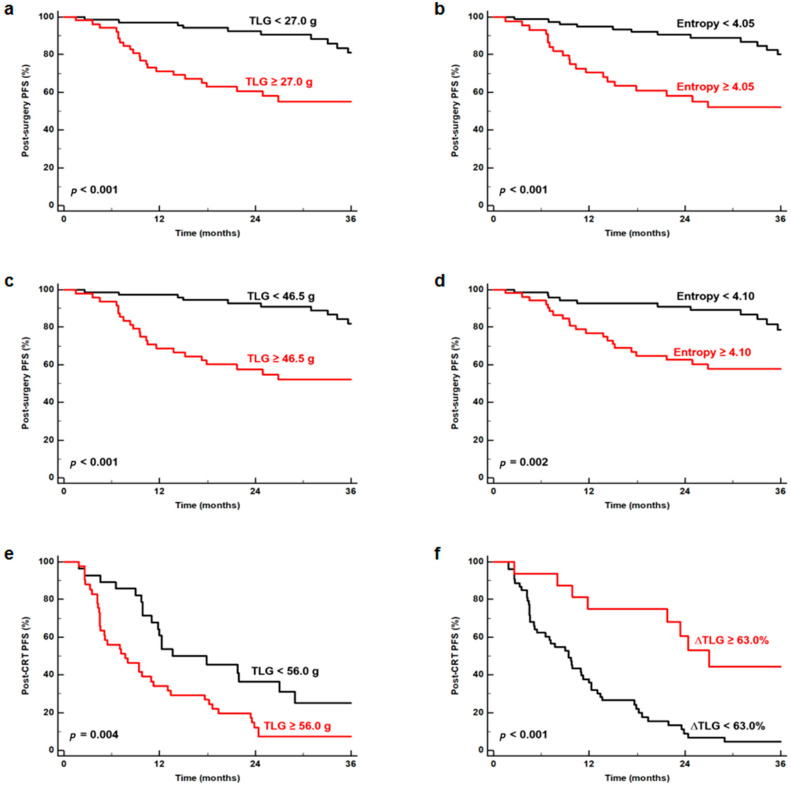
Kaplan–Meier curves for PFS according to TLG (**a**) and entropy (**b**) in the early PET scans and TLG (**c**) and entropy (**d**) in the delayed PET scans in the surgery group (n = 121). Kaplan–Meier curves for PFS according to TLG (**e**) in the early PET scans and ΔTLG (**f**) in the CRT group (n = 69).

In the Kaplan–Meier analysis, TLG and entropy on the early (27.0 g for TLG and 4.05 for entropy) and delayed (46.5 g for TLG and 4.10 for entropy) PET scans in the surgery group and TLG on the early PET scans (56.0 g) and ΔTLG (63.0%) in CRT group were dichotomized according to the specific cut-off values. In the surgery group, the results of the Kaplan–Meier analysis revealed that patients with high TLG and entropy on the early PET scans (TLG, 71.2% vs. 97.1%; entropy, 70.5% vs. 92.8%) and the delayed PET scans (TLG, 68.7% vs. 97.3%; entropy, 76.9% vs. 92.8%) had significantly worse 1-year PFS than those with low values, respectively (*p* < 0.05; Figure 2a–d). In the CRT group, patients with high TLG on the early PET scans also showed significantly worse 1-year PFS (34.1% vs. 64.3%; *p* = 0.004) than those with low values (Figure 2e), whereas significantly better 1-year PFS was observed in patients with high ΔTLG (75.0%) compared with those with a low ΔTLG (37.7%; *p* < 0.001; Figure 2f).

## 4. Discussion

Although the underlying mechanism of the findings of malignant lesions on dual-time-point FDG PET/CT is still not exactly known, previous studies found that the biological characteristics of cancer cells were profoundly related to dual-time-point PET/CT findings [17,24,25]. The FDG uptake of malignant lesions on early PET/CT scans was mainly affected by glucose transporter-1 expression in tumor cells, and the FDG uptake of malignant lesions on delayed PET/CT scans was related to the cell proliferation rate and hexokinase II expression in the tumor cells [17,24,25]. Because the expression of hexokinase II is more particularly increased in cancer cells than in inflammatory cells, the increment of FDG uptake between the early and delayed PET/CT scans was suggested as an imaging biomarker for malignant potential [18,24]. Based on these theoretical backgrounds, several studies have investigated the prognostic significance of dual-time-point FDG PET/CT parameters, especially Δparameters, for predicting the clinical outcomes of patients with NSCLC. In previous studies, the Δmaximum SUV was found to be an independent predictor of the survival of NSCLC patients [17,26,27]. Meanwhile, other studies failed to show any significant association between the Δmaximum SUV and survival [18,19,28]. However, all these previous studies used maximum SUV and Δmaximum SUV as PET imaging parameters, which merely reflect the value of the highest metabolic activity in the cancer lesion and its change [16]. Recently, volumetric parameters and SUV histogram-based parameters have been introduced as imaging parameters, which represent a metabolically active tumor burden and intratumoral metabolic heterogeneity, respectively [21]. These parameters have shown a more significant association with survival than the maximum SUV in various malignant diseases [21,29,30]. However, among the published studies, only a single study demonstrated the prognostic significance of TLG and ΔTLG in dual-time-point PET/CT for predicting the survival of patients with NSCLC [16]. In our study, NSCLC lesions in delayed PET/CT scans showed significantly increased MTV, TLG, and entropy values and decreased energy values, indicating an increased metabolic tumor burden and intratumoral metabolic heterogeneity on the delayed PET/CT images. Moreover, the results of our study revealed the significant prognostic value of MTV, TLG, entropy, ΔMTV, and ΔTLG for predicting PFS, suggesting the role of volumetric parameters and entropy measured from dual-time-point FDG PET/CT as prognostic biomarkers in patients with NSCLC.

One of the major findings of our study was that different dual-time-point PET/CT parameters showed prognostic significance according to the treatment modality. In the CRT group, in addition to TLG in the early PET/CT images, ΔMTV and ΔTLG were found to be independent predictors of PFS. However, in contrast to TLG, which showed an increased risk of disease progression with an increased value, an increase in ΔMTV and ΔTLG was associated with a decreased risk of disease progression. Previous studies have already demonstrated findings similar to our results [16,18]. In a previous study with definitive radiation therapy, a low ΔTLG was associated with a poor local control rate and disease-specific survival [16]. In another study with stereotactic body radiation therapy, an increased Δmaximum SUV was related to lower local recurrence and regional lymph node metastasis [18]. Given that the increment of FDG uptake on delayed PET/CT was related to the proliferation rate and biological activity of tumors, high ΔMTV and ΔTLG could imply the high biological activity of a tumor, which results in being more sensitive to radiotherapy and chemotherapy, thereby, leading to a good prognosis [16,18]. In contrast, in the surgery group, none of the Δparameters showed prognostic significance, and only MTV, TLG, and entropy showed significant association with PFS in multivariate survival analysis. A previous study with surgical resection also demonstrated that the Δmaximum SUV did not have any significant prognostic value for survival [19]. These findings imply that the parameters of a metabolically active tumor burden measured from a single-time-point PET/CT could be suitable PET/CT parameters for predicting prognosis, and the parameters of the biological activity of tumors measured from dual-time-point PET/CT might have a limited prognostic value in the surgery group.

The findings of our study that dual-time-point FDG PET/CT parameters showed different prognostic values between the surgery and CRT groups could provide a clue to understanding the contradictory results of dual-time-point PET/CT studies in patients with NSCLC. Because several studies with dual-time-point PET/CT had enrolled patients with diverse treatment modalities, the prognostic significance of Δparameters could be inconsistent between the studies [17,26,28]. Our results suggest that the parameters of early and delayed PET/CT scans and Δparameters represented different aspects of the tumor characteristics. Therefore, in predicting the prognosis of patients with NSCLC using dual-time-point PET/CT parameters, it might be appropriate to select different imaging parameters according to the treatment modality of the patients. More importantly, our results might be helpful in selecting proper treatment strategies for NSCLC. Stage III NSCLC is considered to have the possibility of being clinically cured [2]. However, although it is generally accepted that multidisciplinary treatment is needed for treating stage III NSCLC, because the boundary between operable and inoperable cancers is not standardized, there is still no unified treatment strategy for patients with stage III NSCLC [2,31]. According to the results of this study, patients with high ΔMTV and ΔTLG could have clinical benefits with a good prognosis when treated with chemotherapy and radiotherapy. Thus, neoadjuvant or radical chemoradiation treatment might be recommended for those patients. Furthermore, several recent clinical trials reported that stereotactic radiotherapy had non-inferior clinical outcomes to surgery in patients with early operable NSCLC, suggesting that radiotherapy could be an alternative treatment to surgery even in early-stage patients [32]. In selecting subjects for stereotactic radiotherapy treatment among patients with NSCLC, the ΔMTV and ΔTLG values might provide information regarding good candidates.

The present study had several inherent limitations. First, this study was retrospectively performed in a single medical center with a relatively small number of patients. Therefore, there might have been a selection bias. Second, because the patients in the CRT group had more advanced NSCLC stages than those in the surgery group, the biological characteristics of NSCLC could be different between the two groups, which might have affected the results of the study. Furthermore, the numbers of patients in both groups were relatively small with an imbalance of the patient numbers between groups. Therefore, further studies are needed to validate our results. Third, PET parameters such as MTV and TLG are dependent on segmentation algorithms for delineating tumor lesions, and the choice of segmentation method could impact the results [33]. Fourth, although acquiring delayed PET/CT images 120 min after the FDG injection was commonly used in previous studies, further studies might be necessary to establish the dual-time-point PET/CT imaging protocol for the best acquisition time [17,18,19,28]. Finally, further investigations based on histopathological and molecular analyses are needed to elucidate the underlying mechanism of the association between dual-time-point PET/CT imaging parameters and disease progression in patients with NSCLC.

## 5. Conclusions

The imaging parameters of dual-time-point FDG PET/CT scans showed different prognostic values according to the treatment modality in patients with NSCLC. For patients treated with surgery, MTV, TLG, and entropy in early and delayed PET/CT images showed a significant association with PFS, with higher values associated with an increased risk of disease progression. For patients treated with chemotherapy and/or radiotherapy, the ΔMTV and ΔTLG values were independent predictors of PFS, with higher values associated with a decreased risk of disease progression. The early and delayed PET/CT image parameters and Δparameters might reflect different aspects of the biological characteristics of NSCLC, and it might be appropriate to select different imaging parameters for predicting the prognosis of NSCLC patients according to the treatment modality.

## Figures and Tables

**Table 1:** Comparison of baseline characteristics between the surgery group (n = 121) and CRT group (n = 69).

Characteristics	All Patients (n = 190)	Surgery Group (n = 121)	CRT Group (n = 69)	*p*-Value
Age (years) *	67 (40–86)	66 (42–85)	70 (40–86)	0.065
Sex				0.099
Men	135 (71.1%)	81 (66.9%)	54 (78.3%)	
Women	55 (28.9%)	40 (33.1%)	15 (21.7%)	
Smoking history				0.014
Yes	127 (67.2%)	73 (60.8%)	54 (78.3%)	
No	62 (32.8%)	47 (39.2%)	15 (21.7%)	
Histopathology				0.523
Adenocarcinoma	113 (59.5%)	75 (62.0%)	38 (55.1%)	
Squamous cell	73 (38.4%)	43 (35.5%)	30 (43.5%)	
Others	4 (2.1%)	3 (2.5%)	1 (1.4%)	
Tumor location				0.911
RUL/RML	74 (38.9%)	45 (37.2%)	29 (42.0%)	
RLL	45 (23.7%)	30 (24.8%)	15 (21.7%)	
LUL	44 (23.2%)	29 (24.0%)	15 (21.7%)	
LLL	27 (14.2%)	17 (14.0%)	10 (14.5%)	
T stage				<0.001
T1–T2	143 (75.3%)	110 (90.9%)	33 (47.8%)	
T3–T4	47 (24.7%)	11 (9.1%)	36 (52.2%)	
N stage				<0.001
N0	110 (57.9%)	91 (75.2%)	19 (27.5%)	
N1	27 (14.2%)	23 (19.0%)	4 (5.8%)	
N2-3	53 (27.9%)	7 (5.8%)	46 (66.7%)	
TNM stage				<0.001
Stage I	82 (43.2%)	78 (64.5%)	4 (5.8%)	
Stage II	40 (21.1%)	29 (24.0%)	11 (15.9%)	
Stage III	68 (35.8%)	14 (11.6%)	54 (78.3%)	
Treatment				
Wedge resection	20 (10.5%)	20 (16.5%)	-	
Lobectomy	93 (48.9%)	93 (76.9%)	-	
Bilobectomy/pneumonectomy	8 (4.2%)	8 (6.6%)	-	
Concurrent chemoradiation	41 (21.6%)	-	41 (59.4%)	
Chemotherapy alone	17 (8.9%)	-	17 (24.6%)	
Radiotherapy alone	11 (5.8%)	-	11 (15.9%)	

Data are presented as the number of patients (%) unless otherwise noted. * Expressed as medians with the range in parentheses. CRT, chemotherapy and/or radiotherapy; LLL, left lower lobe; LUL, left upper lobe; RML, right middle lobe; RLL, right lower lobe; RUL, right upper lobe.

**Table 2:** Comparisons of eight FDG PET/CT parameters of primary lung cancer between early and delayed PET images in all enrolled patients (n = 190).

PET Parameters	Early PET	Delayed PET	*p*-Value	No. of Patients with the Percent Change of the Parameter (ΔPET Parameter) > 0 (%)
Maximum SUV	13.6 (2.6–50.3)	18.3 (2.3–64.0)	<0.001	181 (95.3%)
Mean SUV	8.0 (2.5–24.1)	9.7 (2.0–31.1)	<0.001	158 (83.2%)
MTV	5.0 (0.6–166.4)	6.7 (0.6–179.4)	<0.001	173 (91.1%)
TLG	37.7 (1.5–2090.0)	51.2 (1.5–2796.9)	<0.001	189 (99.5%)
Skewness	0.68 (−0.58–2.04)	0.74 (−0.17–2.64)	0.034	106 (55.8%)
Kurtosis	2.69 (1.00–6.88)	2.76 (1.45–12.08)	0.123	97 (51.1%)
Entropy	3.91 (0.59–5.21)	4.12 (0.44–5.29)	<0.001	138 (72.6%)
Energy	0.07 (0.01–0.76)	0.05 (0.01–0.83)	<0.001	14 (7.4%)

Expressed as medians with range in parentheses. CT, computed tomography; FDG, F-18 fluorodeoxyglucose; MTV, metabolic tumor volume; PET, positron emission tomography; SUV, standardized uptake value; TLG, total lesion glycolysis.

**Table 3:** Univariate survival analysis of PFS in the surgery group (n = 121) and CRT group (n = 69).

Variables		Surgery Group		CRT Group	
		*p*-Value	Hazard Ratio (95% CI)	*p*-Value	Hazard Ratio (95% CI)
Age (1-year increase)		0.366	1.018 (0.980–1.057)	0.562	1.007 (0.984–1.029)
Sex (women vs. men)		0.084	1.997 (0.912–4.374)	0.297	1.425 (0.733–2.773)
Smoking history (no vs. yes)		0.045	2.104 (1.017–4.352)	0.239	1.466 (0.775–2.774)
Histopathology (adenocarcinoma vs.)	Squamous cell	0.375	0.960 (0.254–1.984)	0.455	0.819 (0.485–1.383)
	Others	0.417	1.842 (0.421–8.055)	0.427	1.112 (0.774–2824)
T stage (T1–T2 vs.)	T3–T4	0.048	2.62 (1.006–6.819)	0.049	1.724 (1.003–2.965)
N stage (N0 vs.)	N1	0.003	2.913 (1.428–5.923)	0.006	2.538 (1.320–4.882)
	N2–N3	0.022	3.499 (1.195–10.251)	0.005	5.149 (1.612–16.445)
TNM stage (stage I vs.)	Stage II	0.013	2.493 (1.209–5.141)	0.225	2.656 (0.549–2.845)
	Stage III	0.003	3.963 (1.621–9.691)	0.010	4.591 (1.470–19.708)
Early PET parameters (for 1.0 increase in the parameter value)	Maximum SUV	0.003	1.044 (1.015–1.073)	0.007	1.048 (1.013–1.084)
	Mean SUV	0.002	1.105 (1.039–1.176)	0.006	1.105 (1.028–1.187)
	MTV	<0.001	1.040 (1.021–1.059)	0.006	1.010 (1.003–1.017)
	TLG	<0.001	1.003 (1.002–1.005)	0.004	1.001 (1.000–1.001)
	Skewness	0.331	0.679 (0.311–1.482)	0.737	1.160 (0.488–2.759)
	Kurtosis	0.612	1.083 (0.797–1.471)	0.433	1.127 (0.836–1.519)
	Entropy	<0.001	1.846 (1.295–2.632)	0.235	1.223 (0.877–1.707)
	Energy *	0.017	0.604 (0.400–0.913)	0.067	0.556 (0.311–1.331)
Delayed PET parameters (for 1.0 increase in the parameter value)	Maximum SUV	0.002	1.035 (1.012–1.058)	0.002	1.042 (1.016–1.069)
	Mean SUV	0.004	1.072 (1.023–1.125)	0.006	1.077 (1.021–1.137)
	MTV	<0.001	1.034 (1.012–1.051)	0.228	1.004 (0.998–1.010)
	TLG	<0.001	1.003 (1.001–1.004)	0.168	1.000 (0.999–1.001)
	Skewness	0.514	0.750 (0.316–1.778)	0.759	1.136 (0.504–2.561)
	Kurtosis	0.139	1.187 (0.946–1.491)	0.317	1.175 (0.857–1.612)
	Entropy	0.005	1.766 (1.851–2.631)	0.990	0.999 (0.708–1.412)
	Energy *	0.008	0.374 (0.181–0.776)	0.032	0.385 (0.161–0.923)
ΔPET parameters (for 1.0 increase in the parameter value)	ΔMaximum SUV	0.294	1.006 (0.995–1.018)	0.333	1.018 (0.989–1.035)
	ΔMean SUV	0.300	1.001 (0.995–1.005)	0.384	1.028 (0.981–1.055)
	ΔMTV	0.389	0.999 (0.997–1.002)	0.002	0.989 (0.983–0.996)
	ΔTLG	0.508	0.999 (0.997–1.001)	0.002	0.989 (0.982–0.996)
	ΔSkewness	0.239	0.999 (0.998–1.001)	0.543	0.999 (0.994–1.003)
	ΔKurtosis	0.841	1.000 (0.993–1.008)	0.686	0.998 (0.989–1.007)
	ΔEntropy	0.161	0.993 (0.983–1.003)	0.033	0.982 (0.965–0.999)
	ΔEnergy	0.946	1.000 (0.987–1.015)	0.783	0.996 (0.970–1.023)

* For 0.1 increase in the parameter value. CI, confidence interval; MTV, metabolic tumor volume; PET, positron emission tomography; SUV, standardized uptake value; TLG, total lesion glycolysis.

**Table 4:** Multivariate analysis of dual-time-point PET parameters for predicting PFS in the surgery group (n = 121) after adjustment for age, sex, and TNM stage.

Variables		*p*-Value	Hazard Ratio (95% Confidence Interval)
Early PET parameters (for 1.0 increase in the parameter value)	Maximum SUV	0.260	-
	Mean SUV	0.191	-
	MTV	0.022	1.025 (1.008–1.049)
	TLG	0.024	1.002 (1.001–1.003)
	Entropy	0.039	1.559 (1.076–2.259)
	Energy *	0.177	-
Delayed PET parameters (for 1.0 increase in the parameter value)	Maximum SUV	0.117	-
	Mean SUV	0.149	-
	MTV	0.016	1.024 (1.005–1.044)
	TLG	0.008	1.002 (1.001–1.004)
	Entropy	0.040	1.436 (1.002–2.171)
	Energy *	0.115	-

* For 0.1 increase in the parameter value. MTV, metabolic tumor volume; PET, positron emission tomography; SUV, standardized uptake value; TLG, total lesion glycolysis.

**Table 5:** Multivariate analysis of dual-time-point PET parameters for predicting PFS in CRT group (n = 69) after adjustment for age, sex, and TNM stage.

Variables		*p*-Value	Hazard Ratio (95% Confidence Interval)
Early PET parameters (for 1.0 increase in the parameter value)	Maximum SUV	0.051	-
	Mean SUV	0.087	-
	MTV	0.073	-
	TLG	0.029	1.001 (1.000–1.001)
Delayed PET parameters (for 1.0 increase in the parameter value)	Maximum SUV	0.025	1.123 (1.015–1.242)
	Mean SUV	0.190	-
	Energy *	0.492	-
ΔPET parameters (for 1.0 increase in the parameter value)	ΔMTV	0.010	0.991 (0.984–0.998)
	ΔTLG	0.007	0.991 (0.984–0.998)
	ΔEntropy	0.289	-

* For 0.1 increase in the parameter value. MTV, metabolic tumor volume; PET, positron emission tomography; SUV, standardized uptake value; TLG, total lesion glycolysis.

## Data Availability

The datasets used and/or analyzed during the current study are available from the corresponding authors on reasonable request.

## Supplementary Materials

The following supporting information can be downloaded at: https://www.mdpi.com/article/10.3390/tomography8020087/s1, Table S1: Definition of SUV histogram-based first-order PET parameters.
