# Supplementary material for: Different Prognostic Values of Dual-Time-Point FDG PET/CT Imaging Features According to Treatment Modality in Patients with Non-Small Cell Lung Cancer
Source: Tomography. 2022 Apr 8;8(2):1066–78. doi: 10.3390/tomography8020087 (PMC9028882; doi:10.3390/tomography8020087)
Supplement: Supplementary file 1 [file tomography-08-00087-s001.zip › tomography-1662694-supplementary.pdf]

Table S1. Definition of SUV histogram-based first-order PET parameters.

PET parameters	Definition
Skewness	Asymmetry of the grey-level distribution in the histogram<br>Skewness = $[\frac{1}{N}\sum_i (I(i) - Iavg)^3]$ / $[\frac{1}{N}\sum_i (I(i) - Iavg)^2]^{\frac{3}{2}}$<br>I(i) corresponds to the number of voxels with intensity i, N the total number of voxels in the volume of interest, and Iavg the average of grey-levels
Kurtosis	Shape of the grey-level distribution in the histogram relative to a normal distribution<br>Kurtosis = $[\frac{1}{N}\sum_i (I(i) - Iavg)^4]$ / $[\frac{1}{N}\sum_i (I(i) - Iavg)^2]^2$<br>I(i) corresponds to the number of voxels with intensity i, N the total number of voxels in the volume of interest, and Iavg the average of grey-levels
Entropy	Randomness of the grey-level distribution in the histogram<br>Entropy = $-\sum_i p(i) \log_2(p(i) + \varepsilon)$<br>P(i) is the probability of occurrence of voxels with intensity i and ε = 2e -16
Energy	Uniformity of the grey-level distribution in the histogram<br>Energy = $\sum_i p(i)^2$<br>P(i) is the probability of occurrence of voxels with intensity i
